# Quantitative characterization of the auxin-inducible degron: a guide for dynamic protein depletion in single yeast cells

**DOI:** 10.1038/s41598-017-04791-6

**Published:** 2017-07-05

**Authors:** Alexandros Papagiannakis, Janeska J de Jonge, Zheng Zhang, Matthias Heinemann

**Affiliations:** Molecular Systems Biology, Groningen Biomolecular Sciences and Biotechnology Institute, University of Groningen, Nijenborgh 4, 9747 AG Groningen, The Netherlands

## Abstract

Perturbations are essential for the interrogation of biological systems. The auxin-inducible degron harbors great potential for dynamic protein depletion in yeast. Here, we thoroughly and quantitatively characterize the auxin-inducible degron in single yeast cells. We show that an auxin concentration of 0.25 mM is necessary for fast and uniform protein depletion between single cells, and that in mother cells proteins are depleted faster than their daughters. Although, protein recovery starts immediately after removal of auxin, it takes multiple generations before equilibrium is reached between protein synthesis and dilution, which is when the original protein levels are restored. Further, we found that blue light, used for GFP excitation, together with auxin results in growth defects, caused by the photo-destruction of auxin to its toxic derivatives, which can be avoided if indole-free auxin substitutes are used. Our work provides guidelines for the successful combination of microscopy, microfluidics and the auxin-inducible degron, offering the yeast community an unprecedented tool for dynamic perturbations on the single cell level.

## Introduction

Perturbations are necessary tools for the investigation of biological systems^1^. Next to static gene deletions, dynamic and reversible perturbations of protein levels are necessary to investigate pathways and their temporal dynamics, resulting either from cell cycle activity, stochasticity in gene expression, or responses to environmental stimuli. While the dynamic down-regulation of protein synthesis can be accomplished on the transcriptional or posttranscriptional level, both perturbations have only an effect on newly expressed proteins. Due to the typically slow protein turnover and growth-related dilution rates^2^, these perturbations are severely limited in terms of their dynamics. Perturbations directly on the protein level are thus desirable.

In yeast, temperature-sensitive mutants^3^ have traditionally been used to deplete proteins, and were followed up by the heat-inducible degron^4^. Although both methods result in targeted protein inactivation or depletion, they require a change in temperature, which could cause global effects on cellular physiology^5^. Novel perturbation methods use different means of induction. A photo-sensitive degron, activated by blue light, was developed by fusing the cODC C-terminal degron to the light oxygen voltage 2 (LOV2) photoreceptor domain from *Arabidopsis thaliana* ^6, 7^, exhibiting fast protein depletion dynamics (i.e. 12 minutes). However, this light inducible degron requires constant blue light illumination, which is known to induce cellular photo-toxicity^8^, thus significantly reducing cell viability and prohibiting its combination with the most widely used fluorescent protein, in dynamic protein depletion experiments.

Alternatively, the auxin-inducible degron (AID), originating from plants, was successfully assembled in yeast and mammalian cells^9^, where it was used for the rapid (<1 hour) and targeted depletion of proteins tagged with the degron domain. The plant hormone auxin (indole-3-acetic acid; IAA) mediates the interaction between the tagged protein and the plant F-box protein TIR1, which forms an active complex with the conserved E3 ubiquitin ligase components, resulting in the poly-ubiquitylation of the degron domain and the proteasomal degradation of the tagged protein^9^. Truncated versions of the degron sequence^10, 11^, combined with various selection markers, epitope tags (i.e. c-myc, HA, FLAG, GFP)^11^ and flanking motifs^12^ have increased the versatility of the AID system and its applicability in yeast.

While the system has been used in several yeast studies, for instance to study chromosomal structure and dynamics^13–17^, DNA replication, cytokinesis^18^, autophagy^19, 20^, gametogenesis^21^, ribosomal synthesis^22^ and exocytosis^23^, it turns out that the potential off-target effects of the AID system and of the plant hormone auxin in yeast have only been tested qualitatively with spot assays^9, 11, 24, 25^. Furthermore, until today, the AID system has only been characterized on the yeast population level. In principle, the AID system should be a great tool for single cell studies, especially combined with the recently developed microfluidics devices^26–32^, where yeast cells are grown under the constant perfusion of nutrients. In microfluidics, protein degradation signals (e.g. auxin) can be added or removed from the system simply by a switch of the perfused medium. No additional cell manipulation, including centrifugation or washing, is required, thus avoiding potential stresses or technically induced phenotypic variability. Despite the apparent advantages of single cell technologies in perturbation experiments, the potential of the AID system in combination with microfluidics remains unexplored.

Here, we performed an in-depth characterization of the AID system in single yeast cells. We show that the auxin-inducible degradation can be successfully applied in microfluidics for fast and targeted protein depletion. Higher auxin concentrations result in faster protein depletion with lower cell-to-cell variability. The AID system has no effect on growth rate when auxin concentrations up to 0.1 mM are applied. However, blue light, used for GFP excitation, in combination with auxin (indole acetic acid) causes growth defects, which can be avoided by using naphthalene-acetic acid (NAA, a synthetic auxin substitute), which also induces protein degradation. Upon removal of the plant hormone, it takes several generations until the equilibrium between protein synthesis and dilution is reached again, meaning that the recovery of the original protein levels is slow. In two case studies, we show the strengths and limitations of the AID system. With microfluidics technology becoming widely accessible, we anticipate that our thorough characterization of the AID system and establishment of its use on the single cell level will be of significant value for the yeast community, seeking for tools to perturb proteins and pathways in a targeted manner.

## Results

### Protein depletion dynamics upon the addition of different auxin concentrations

**Figure 1:**
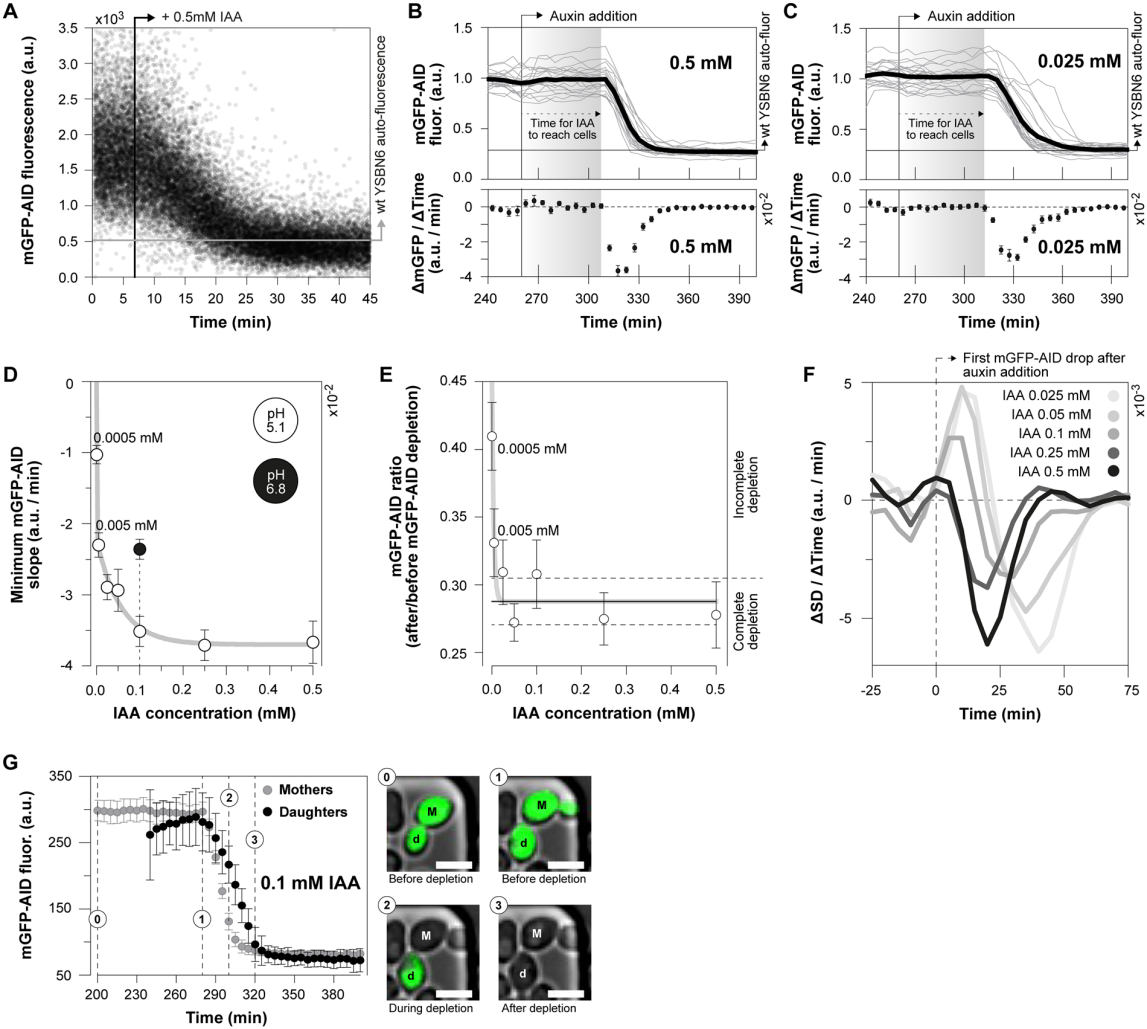
Auxin concentration-dependent dynamics of targeted protein depletion. (**A**) The mGFP-AID fluorescence continuously measured using flow cytometry. 0.5 mM of auxin were added at 06:50 mm:ss. Each data point corresponds to a single cell. (**B**,**C**) The average mGFP-AID depletion dynamics upon the addition of (**B**) 0.5 mM (20 cells) and (**C**) 0.025 mM (22 cells) auxin in the microfluidic device. Top figures: single cell (grey lines) and their average (black line) mGFP-AID trajectories. Each single cell trajectory was normalized by dividing over the mean mGFP-AID signal from 0 to 240 minutes. Bottom figures: average rate (ΔmGFP/ΔTime) of mGFP-AID depletion (error bars: SEM). The levels of yeast auto-fluorescence were divided over the mGFP-AID levels prior the addition of auxin to indicate the point of complete protein depletion. When we use the syringe pump for the perfusion of medium through the chip, it takes 50 ± 5 minutes for auxin to reach the cells after the switch. This lag time is indicated. (**D**) The minimum mGFP-AID slope (as in Fig. 1B‚C), or the maximum rate of mGFP-AID depletion, plotted against the auxin concentration (error bars: SEM). For an auxin concentration of 0.1 mM the rate of protein depletion was measured at pH 5.1 (white marker) and pH 6.8 (black marker). A two-phase exponential decay function was fitted to the white markers (y-intercept set to zero). Raw data, including the numbers of analyzed cells, are presented in Fig. S2A and B. **(E)** The completeness of protein depletion: the average mGFP-AID signal after the addition of auxin (500–740 min – as in Fig. 1B,C) was divided by the same signal before the addition of auxin (0–240 min – as in Fig. 1B,C) for each single cell and auxin concentration. The average ratios are presented and their 95% CI (error bars) reflect cell-to-cell variability. The horizontal lines correspond to the range of complete protein depletion (mean: solid line, 95% CI: dashed lines), estimated non-parametrically (bootstrapping, 100 iterations) by dividing the auto-fluorescence of wild type yeast (13 non-fluorescent cells) by the average mGFP-AID fluorescence (15 cells prior to depletion). A log(dose) response curve with a variable slope was fitted to the data (y-intercept was set to 1, base set to 0.288 – average ratio for complete depletion). (**F**) The change of the standard deviation of the mGFP-AID signal per time interval and auxin concentration reports the uniformity of the response to auxin. Positive slopes denote an increasing standard deviation and a variable response. Negative slopes denote a decrease in cell-to-cell mGFP-AID variability. (**G**) Comparison of the average mGFP-AID depletion dynamics (error bars: 95% CI) between yeast mothers (21 cells) and daughters (7 cells). Microscopy images display their different mGFP-AID depletion (scale 5 μm, same contrast applied in all images). See also Movie S1.

To investigate the auxin inducible protein degradation dynamics, across a wide range of auxin concentrations (from 0.5 μΜ to 0.5 mM) in single *Saccharomyces cerevisiae* cells, we used a monomeric GFP variant tagged with the truncated degron sequence AID^71–114^ (mGFP-AID), in cells expressing the TIR1 F-box protein from *Oryza sativa* (Os-TIR1) (Table S1). Cells were grown in minimal medium^33^ with 10 gL^−1^ glucose. First, mimicking population-level depletion experiments, we used flow-cytometry and continuously followed the cellular fluorescence upon the addition of 0.5 mM auxin. Here, in agreement with previous immunoblotting experiments^9^, which reported a time of 15 to 45 minutes for complete protein depletion upon the addition of the same auxin concentration, we found the protein to be fully depleted 25 minutes after the addition of auxin (Fig. 1A).

To measure the protein depletion dynamics in single cells, we used microfluidics combined with fluorescence time-lapse microscopy (Fig. S1A). Yeast cells were cultivated in the microfluidic dissection platform^26, 27^ and their mGFP-AID fluorescence intensities were recorded every 5 minutes, before and after the addition of auxin (Fig. 1B,C, upper panels). The average rate of protein depletion was determined from the single cell mGFP-AID trajectories and their first derivatives, at different auxin concentrations (Fig. 1B,C, lower panels). Focusing on the lowest negative derivative, corresponding to the highest rate of mGFP-AID depletion (e.g. lowest point in Fig. 1B,C, lower panels), we found that the rate of protein depletion correlated with the applied auxin concentration (Fig. 1D). The maximum depletion rate was achieved for auxin concentrations equal or higher than 0.25 mM. For the same concentration of auxin (0.1 mM), growth medium with increased pH (from pH 5.1 to 6.8) lead to lower mGFP-AID depletion rates (Fig. 1D), possibly caused by the de-protonation of auxin^34^, and thus its reduced uptake by yeast cells^35^. Consistent with the very low rates of protein depletion at auxin concentrations below 0.005 mM (Fig. 1D), we observed incomplete degradation of the protein (Fig. 1E).

Next, we assessed the intercellular variability in the auxin-induced protein degradation. Inspection of single-cell mGFP-AID trajectories showed that high auxin concentrations (e.g. 0.5 mM – Fig. 1B) result in uniform protein depletion dynamics as compared to lower auxin concentrations, where cells respond at different times (e.g. 0.025 mM – Fig. 1C). As a quantitative measure for the intercellular variability we used the standard deviation of the average mGFP-AID signal at each time point, determined for each auxin concentration. An increase in the standard deviation (positive derivative) signifies a variability increase upon the addition of auxin (as in Fig. 1C). Adversely, a decrease (negative derivative) reports decreased variability and thus a uniform response to the plant hormone (as in Fig. 1B). Here, we found that the addition of 0.25 mM auxin or higher reduces the intercellular variability in a dose-dependent manner, whereas lower auxin concentrations (≤0.1 mM) lead to increased variability (Fig. 1F). In our single-cell experiments, we also found that the daughter cells, born just prior to the auxin appearance in the medium, show significantly slower (approx. 20 min later) mGFP-AID depletion when compared to their mothers (Fig. 1G, Movie S1).

These experiments reveal the effective range of the AID system: High auxin concentrations (≥0.25 mM) result in maximal depletion rate, and a uniform response, whereas low auxin concentrations (<0.25 mM) result in slower concentration-dependent rates of depletion with increased cell-to-cell variability. Furthermore, with the extracellular pH influencing the protonation of auxin, the pH of the medium has an effect on the cellular auxin uptake and thus the protein depletion dynamics.

### Auxin causes growth defects when combined with blue light used for GFP excitation

Next to the dynamic nature, orthogonality is an equally important characteristic of perturbation systems. With the most distinguishable and easily observable manifestation of lack of orthogonality being growth defects, here, we investigated whether and if yes, in how far, the different elements of the AID system affect growth, both in yeast populations (batch cultures) and in single cells grown in the microfluidic device.

**Figure 2:**
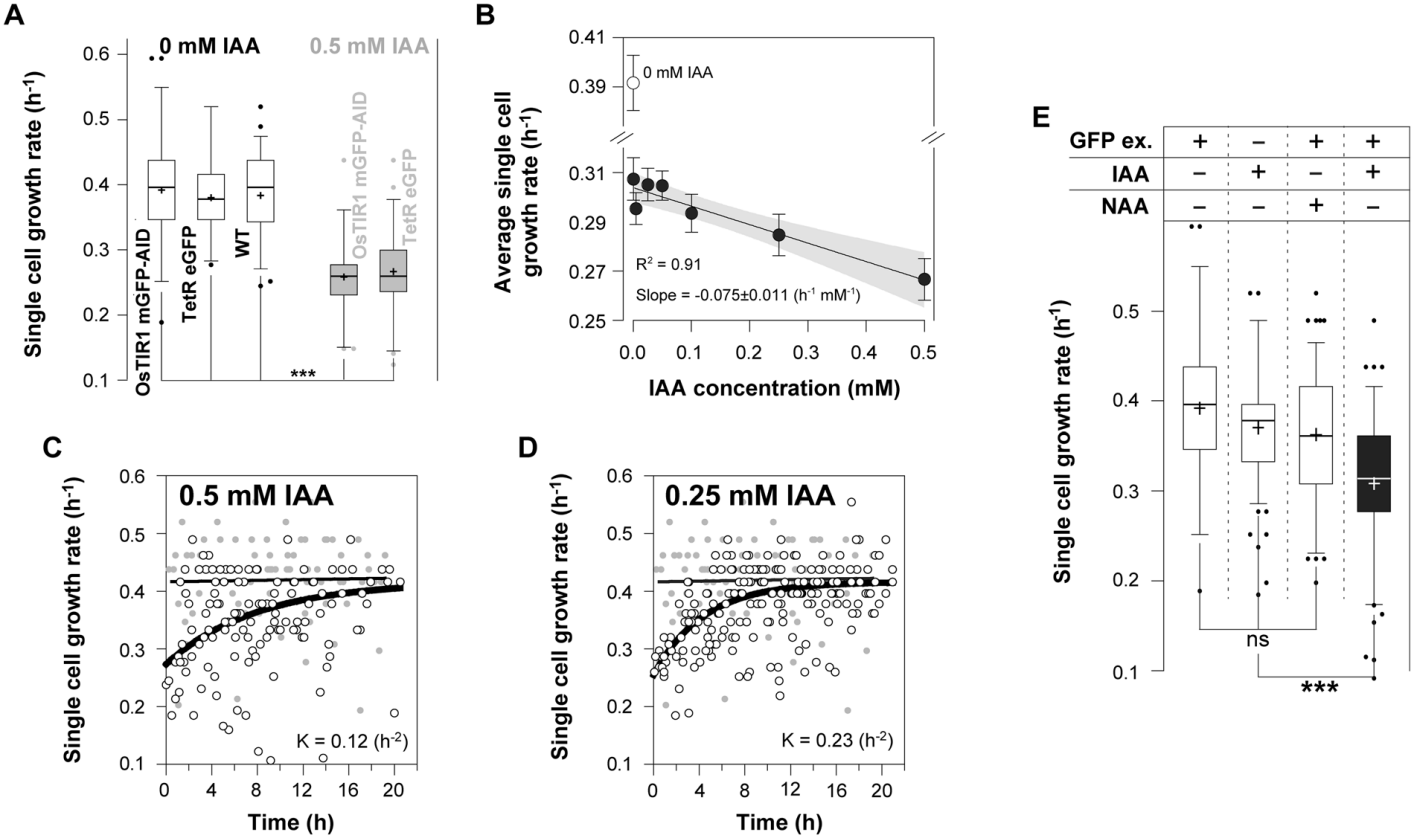
Auxin causes concentration-dependent and reversible growth defects, especially when combined with blue light for GFP excitation. (**A**) In order to measure potential effects of OsTIR1 or GFP expression on growth, we performed single cell experiments with cells expressing the plant F-box protein (YSBN6.G2J – Table S1), or eGFP via a tetracycline inducible system (YSBN6.C6B – Table S1), and measured their growth rates in the presence (grey boxes) and absence (white boxes) of auxin (error bars: 5–95 percentile, cross: mean). From left to right: 51 budding cycles from 37 single cells, 31 from 30, 50 from 9, 43 from 26 and 50 budding cycles from 19 single cells. The single cell growth rates (GR_sc_) were estimated on the basis of the single cell doubling time (DT_sc_), or the time between two consecutive budding events $(G{R}_{sc}=\frac{ln(2)}{D{T}_{sc}})\,$. The addition of 0.5 mM of auxin in the microfluidics device significantly slows down growth independently of the OsTIR1 expression (Kruskal-Wallis test, Dunns post-test to compare all pairs of data, p < 0.001 ***). (**B**) The average growth rate of yeast was estimated in the presence of auxin at different concentrations, as well as in the absence of auxin on the single-cell level (error bars: SEM). From left to right: 54 budding cycles from 20 single cells, 57 from 27, 51 from 14, 50 from 16, 41 from 14, 51 from 19 and 50 budding cycles from 19 single cells. (**C**,**D**) Growth rate recovery after the removal of auxin (at 0 hours). One-phase exponential association functions were fitted to the recovering growth rates (K: rate constant). The plateau was set to the average growth rate of unperturbed wild type cells, grown for the same time in the microfluidic device (grey markers). A linear regression was fitted to the control data to illustrate unperturbed and steady state growth on high (10 gL^−1^) glucose, in the microfluidic device. Data from (**C**) 195 budding cycles and 24 single cells, or (**D**) 126 budding cycles from 20 single cells are included. (**E**) The single cell growth rates of OsTIR1 expressing cells (error bars: 5–95 percentile, cross: mean), grown and monitored in the microfluidics, together with (+) or without (−) GFP excitation, with IAA (0.1 mM) or NAA (0.1 mM). From left to right: 51 budding cycles from 37 single cells, 117 from 39, 157 from 30 and 50 budding cycles from 16 single cells. IAA significantly reduces growth only in combination with GFP excitation (Kruskal-Wallis test, Dunns post-test to compare all pairs of data, p < 0.001 ***).

First, we found that the expression of the plant F-box protein (TIR1) alone, in the absence of auxin, did not affect the growth of yeast (Fig. 2A), indicating that neither the expression of the OsTIR1, nor an eventual degradation of untargeted (non-tagged) proteins, causes a growth rate effect. However, in the microfluidic setup, upon the addition of auxin we found a strong growth defect (Fig. 2A) with concentrations as low as 0.5 μM already causing a 35% reduction in growth rate (Fig. 2B). By removing the auxin from the microfluidic device, the growth rate recovered within 12 hours (Fig. 2C,D). Thus, while the OsTIR1 complex has no effect on growth, we found that auxin – in microfluidic experiments with microscopic observation – slows down the growth of yeast cells. Notably, we found the growth defects to be less pronounced in batch cultures, where only auxin concentrations higher than 0.1 mM lead to slower growth (Fig. S3).

In an attempt to explain the cause of this growth rate defect, we focused on the technical differences between experiments performed in microfluidics (Fig. 2A,B) and those performed in batch cultures (Fig. S3). First, in exponentially growing batch cultures the daughter cells constitute a significant fraction (approx. 50%) of the measured population and thus contribute significantly to the averaged population characteristics (e.g. growth rate). In contrast, in the microfluidic device we only focus on mother cells, because the daughters are constantly washed away. If auxin affects the growth of mothers differently to daughter cells, similarly to what holds for the protein depletion dynamics (Fig. 1G, Movie S1), such difference could account for the observed discrepancy between our population and single cell measurements. However, when we analyzed the doubling times of mothers in comparison to the few daughters, which were retained in the microfluidics device, we found them to be equally affected (Fig. S4).

Second, in the microfluidic device we regularly (every 5 min) exposed the cells to blue light for the excitation of GFP, which was not the case in the batch culture. In order to test if the blue light causes the pronounced growth rate reduction when auxin is present in microfluidics, we measured the effect of each factor (GFP excitation and auxin) on growth separately and in combination. We found that blue light or 0.1 mM auxin alone caused no significant growth rate reduction (Fig. 2E), similarly to our population level measurements (Fig. S3). However, the combination of the plant hormone with blue light causes a pronounced growth rate reduction, even at low auxin concentrations (e.g. 0.5 μM) (Fig. 2B and E).

Indole-3-acetic acid (auxin) is known to be sensitive to blue light. Blue light accelerates the oxidative de-carboxylation of indole-3-acetic acid to methylene-oxindole and^36^ which was previously shown to cause cytotoxicity^37^, and we hypothesized that this causes the growth rate reduction in our microscopy experiments. Indeed, when we used naphthalene-acetic acid (0.1 mM), a synthetic, indole-free substitute of auxin, to deplete the targeted protein (mGFP-AID) under frequent (i.e. every 5 min) GFP excitation, we did not observe any significant effect on growth (Fig. 2E). When we measured the dynamics of protein depletion with NAA (Fig. S5A and B) we found them to be similar to the ones with IAA for concentrations equal or above 0.05 mM (Fig. S5C). Thus, we recommend the use of NAA for the protein depletion in microscopy experiments with GFP excitation.

### Protein recovery dynamics upon the removal of auxin

**Figure 3:**
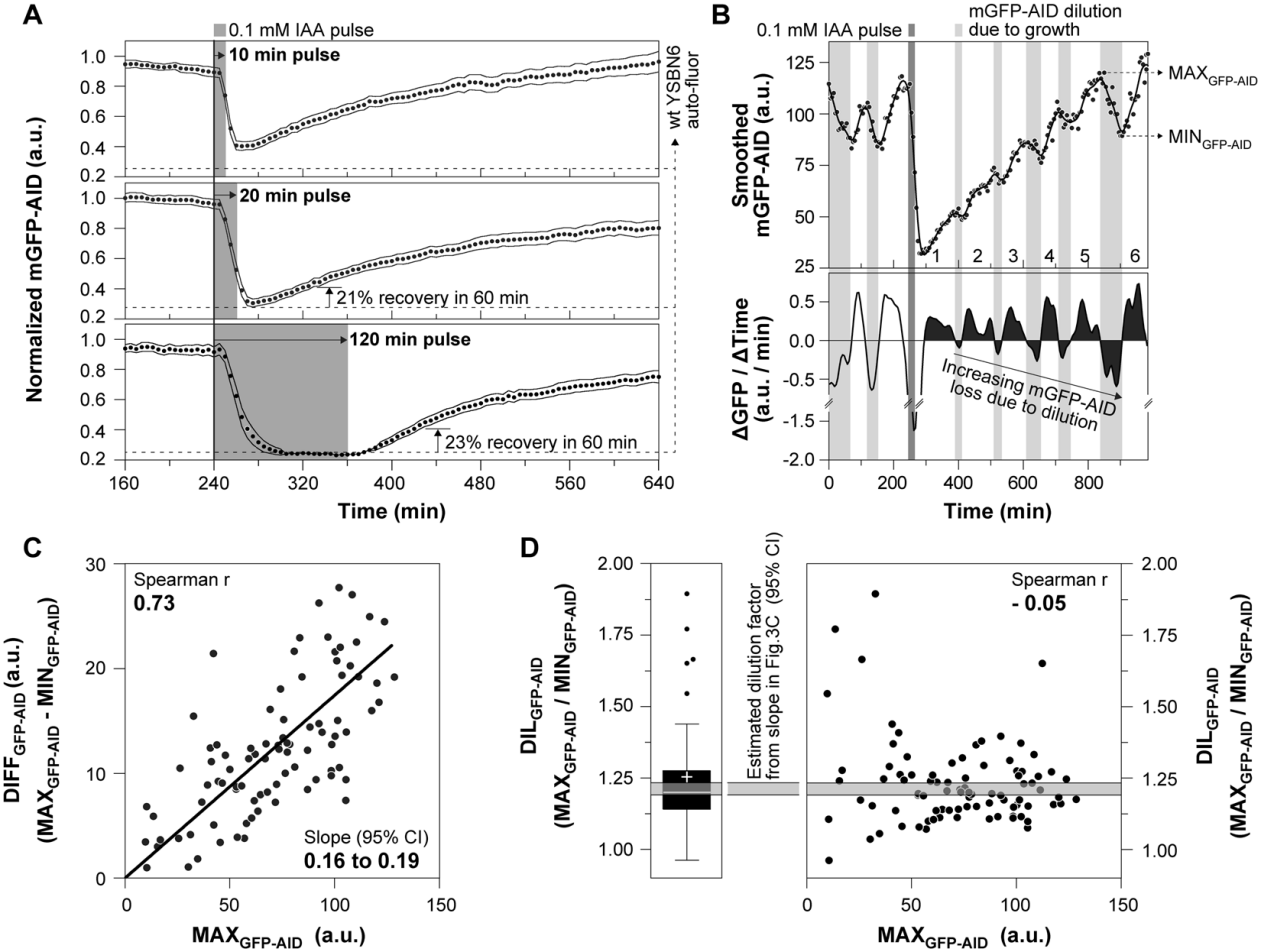
After auxin removal, protein recovery is complete when protein synthesis and dilution reach a new equilibrium, and thus takes several generations. (**A**) Auxin pulses of 10 min (top figure – 36 single cells), 20 min (middle figure – 36 single cells) and 120 min (bottom figure – 38 single cells) were provided to estimate the protein recovery dynamics after complete or partial protein depletion, in three separate experiments (black markers: averages, error bands: 95% CI). Each single cell trajectory was normalized by dividing over the mean mGFP-AID signal from 0 to 240 minutes, as in Fig. 1B,C. The levels of yeast auto-fluorescence, corresponding to complete protein depletion, were estimated by multiplying the mGFP-AID signal at the time of auxin addition (240 min) by the complete depletion factor (folds of fluorescence drop during complete depletion), estimated in Fig. 1E. The percentage of protein recovery at 60 min after auxin removal is presented for the 20 min and 120 min pulse experiments. (**B**) Raw single cell mGFP-AID measurements before, during and after the addition of 20 min auxin pulse (single cell from Fig. 3A – middle plot). A smoothing spline was fitted to the data and was used to estimate the first derivative of the mGFP-AID dynamics (ΔGFP/ΔTime). The integrals of the positive or negative derivatives during protein recovery (after auxin removal), indicating the total amount of protein synthesized or diluted in the yeast mother cell per division cycle, are shaded black. Each number (from 1 to 5) corresponds to one budding and thus division cycle after auxin removal, as observed under the microscope, in the DIC channel. (**C**) The maximum mGFP-AID levels just prior to dilution (*MAX*_*GFP*−*AID*_), corresponding to the peaks of the oscillating mGFP-AID signal after auxin removal (as in Fig. 3B – top), strongly and positively correlate with the drop in the mGFP-AID fluorescence (*DIFF*_*GFP*−*AID*_), corresponding to the signal at the peak (*MAX*_*GFP*−*AID*_) minus the signal at the next trough (*MIN*_*GFP*−*AID*_). A linear regression was fitted to the data and the 95% CI of its slope (*SLOPE*) were estimated non-parametrically using bootstrapping. Data from 91 mGFP-AID oscillations, from 19 single cells are presented. (**D**) The slope if the fitted linear regression in Fig. 3C was used to estimate the factor of protein dilution (*DIL*_*GFP*−*AID*_) per cell cycle. Specifically we know: $DI{L}_{GFP-AID}=\frac{MA{X}_{GFP-AID}}{MI{N}_{GFP-AID}}$. We also know: $SLOPE=\frac{DIF{F}_{GFP-AID}}{MA{X}_{GFP-AID}}=\frac{MA{X}_{GFP-AID}\,-MI{N}_{GFP-AID}}{MA{X}_{GFP-AID}}=1-\frac{MI{N}_{GFP-AID}}{MA{X}_{GFP-AID}}$. Thus: $DI{L}_{GFP-AID}=\frac{1}{1-SLOPE}$. The estimated dilution factor (grey shaded area – 95% CI) is consistent with the experimentally determined dilution factor (Tukey box plot, cross: mean) for each mGFP-AID oscillation during recovery. We found no correlation between the MAX_GFP−AID_ levels and the dilution factor, which confirms a constant mGFP-AID factor of dilution across cell divisions. Data from 91 mGFP-AID oscillations, from 19 single cells are presented, as in Fig. 3C.

Next, we assessed the reversibility of the AID system. After the removal of auxin from the environment, protein degradation is expected to stop, and the concentration of the protein should recover to its normal levels due to re-synthesis. To assess this, we made use of the medium-switch possibility provided by the microfluidic device in combination with a finely controlled perfusion system (Fig. S1B), capable of achieving a complete switch between media compositions within 3–4 minutes (Fig. S1C), well below our sampling period (5 minutes). We performed three experiments, where we exposed cells for 10, 20 and 120 minutes to auxin. First, we found that an auxin pulse of at least 20 minutes is required for complete protein depletion (Fig. 3A). Second, in all three experiments, the fully or partially depleted mGFP-AID started recovering almost immediately after switching back to medium without auxin (Fig. 3A). These results demonstrate the reversibility of the auxin-inducible degron. Still, we were surprised to find that it took at least 300 minutes for mGFP-AID to reach the levels prior to depletion.

In order to better understand the mGFP-AID recovery dynamics and explain the long time required for complete protein recovery, we zoomed into single cells. Here, we found that the protein recovery occurs in waves of protein concentration increase and protein dilution with each cell cycle (Fig. 3B). We found a strong positive linear correlation (Fig. 3C) between the mGFP intensity (MAX_GFP−AID_, as in Fig. 3B) and the mGFP intensity drop (MAX_GFP−AID_ – MIN_GFP−AID_, as in Fig. 3B), which suggested a constant dilution factor due to cell growth and division, which - using the slope of the correlation (Fig. 3C) - we estimated to be 1.2 (Fig. 3D – grey band, DIL_GFP−AID_). As we found this factor to be in agreement with the experimentally estimated dilution in single cells (Fig. 3D – box-plot and scatter-plot), we concluded that the drops in GFP intensity are because of dilution due to growth.

Thus, while the AID system is fully reversible, as indicated by the almost immediate protein increase after the auxin removal, it takes several divisions until the original protein levels are reached. After removal of auxin, the protein concentration increases (due to protein synthesis), and so does the amount of protein diluted due to cell growth per cell cycle, until these two processes reach an equilibrium. At this point, the original protein level is achieved. The slow protein recovery might represent a limitation of the AID system, especially when studying fast processes where complete protein recovery is required to reverse the conditional depletion phenotype. However, 20% of original protein levels are recovered within one hour after the removal of the plant hormone (Fig. 3A – 20 min and 120 min pulse). If such a partial recovery is enough to reverse the conditional depletion phenotype, then the AID may also be used to reversibly perturb processes within a single cell generation. In all cases, the interesting dynamics of protein recovery might also harbor information on the processes of protein synthesis and dilution during cell division.

### Using the AID system to generate growth-related depletion phenotypes

Next, we set to test the AID system in two single-cell case studies. In the first one, we depleted a non-essential protein, the Gcn5 acetyltransferase. Gcn5 is an N-acetyltransferase, activator and catalytic subunit of the SAGA (Spt-Ada-Gcn5-Acetyltrasnderase) transcriptional co-activator^38^. Gcn5 transfers acetyl groups from acetyl-CoA to the core of H3 and H4 histones, activating the expression of growth related genes in an acetyl-CoA concentration-dependent manner^39, 40^. Deletion of the Gcn5 acetyltransferase has been shown to reduce the growth rate of yeast by approximately 60%^39, 41^.

**Figure 4:**
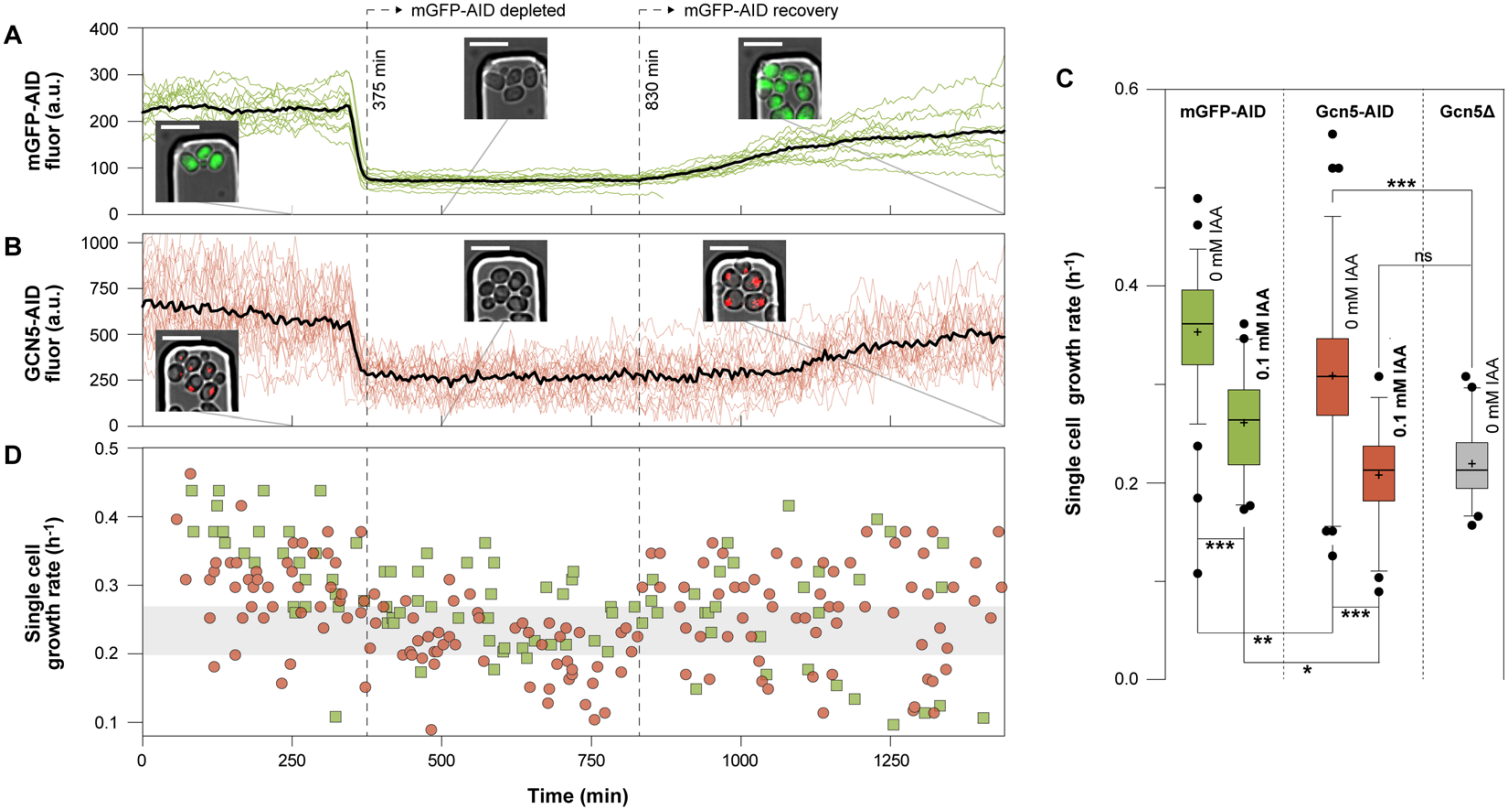
The auxin-induced growth defects can be discriminated from the growth-related Gcn5 depletion phenotype. (**A**) Control cells expressing mGFP-AID were mixed with (**B**) cells expressing Gcn5-mCherry-AID and grown in the same microfluidic device on 10 gL^−1^ glucose. Upon the addition of auxin (0.1 mM), mGFP-AID and the nuclear Gcn5-mCherry-AID were depleted at the same time (375 min). The recovery dynamics of the two proteins after auxin removal (at 830 min) are also visible. The black trajectories correspond to the average mGFP-AID (14 cells) and Gcn5-mCherry-AID (23 cells) signals. The colored trajectories correspond to the single cell signals. Microscopy images confirm the localization and levels of the fluorescent proteins (scale 10 μm) (**C**) The single cell growth rates in the absence (0 mM IAA) and after the addition (0.1 mM IAA) of auxin, of mGFP-AID (green boxes), Gcn5-mCherry-AID (red boxes) and *Gcn5Δ* cells (grey boxes) growin in minimal medium with high (10 gL^−1^) glucose (error bars: 5–95 percentile, cross: mean). From left to right: 79 budding cycles from 34 cells, 40 from 14, 76 from 41, 52 from 23, 40 from 17 single cells. (Kruskal-Wallis test, Dunns post-test to compare all pairs of data, p < 0.001 ***, p < 0.01 **, p < 0.05 *, p > 0.05 ns). (**D**) The single cell growth rates at different time points are plotted for each yeast strain (green squares: mGFP-AID, red circles: Gcn5-mCherry-AID).

Here, we used the AID system to dynamically deplete Gcn5. In the microfluidics device, we simultaneously loaded cells expressing a version of Gcn5 that was tagged with mCherry and the degron sequence AID^71–114^ (Gcn5-mCherry-AID), and control cells expressing mGFP-AID. The two strains were easy to distinguish due to their distinct fluorescent tags (nuclear mCherry, and cytoplasmic mGFP, respectively – Fig. 4A,B).

First, we tested if we could use the control strain, to indicate the Gcn5 depletion dynamics. After the addition of auxin (0.1 mM), we found that the cytoplasmic mGFP-AID and the nuclear Gcn5-mCherry-AID fluorescence disappeared simultaneously (Fig. 4A,B). Thus, the depletion dynamics of the two proteins are identical. Our results demonstrate that once established, it is possible to use a control strain (e.g. mGFP-AID) in parallel to indicate the depletion dynamics of a targeted protein that is not fluorescently labeled. This increases the availability of fluorescent proteins, which can be used to monitor other than the targeted proteins during conditional depletion, and thus increases the versatility of the AID system on the single cell level.

Next, also using the control strain (mGFP-AID), we tested if we could discriminate between off-target growth defects and those specific to the conditional depletion of the N-acetyltransferase. Before the addition of auxin, we found the Gcn5-mCherry-AID cells to exhibit significantly (Kruskal-Wallis test, Dunns post-test, p < 0.01 **) slower growth, when compared to the control mGFP-AID cells (Fig. 4C – 0 mM), indicating either a basal level of Gcn5 depletion or an effect of the degron tag on the activity of the protein. Immediately after the auxin addition, the Gcn5-mCherry-AID depleted cells exhibited a growth rate similar to the Gcn5 deletion strain (Kruskal-Wallis test, Dunns post-test, p > 0.05 ns) and significantly lower when compared to the control cells (Kruskal-Wallis test, Dunns post-test, p < 0.05*), reflecting the unspecific growth defects of auxin when combined with GFP measurements. These results demonstrate that with the parallel use of a control strain (e.g. mGFP-AID) it is possible to decipher between specific and unspecific growth defects (Fig. 4C,D). After removal of auxin, the growth rate recovered (Fig. 4D) and the depleted proteins slowly re-appeared in their subcellular compartments (Fig. 4A,B).

### Using the AID system to deplete essential proteins and generate lethal phenotypes

In a second case study, we used the AID system to dynamically deplete the essential cell cycle proteins Cdc28 and Cdc14. Cdc28 is the cyclin dependent kinase (CDK) of yeast controlling the G1/S transition^42^, the periodic cell cycle transcription and chromosome dynamics in response to periodic waves of early and late cyclins, as well as filamentous growth in response to nutrients and storage carbon metabolism^43^. Cdc14 is a phosphatase and essential activator of the anaphase promoting complex (APC)^44^. It is necessary for the proteasomal degradation of late cyclins and the de-phosphorylation of the CDK targets for the completion of the cell cycle program and the entry into G1 phase. Conditional depletion of Cdc28 is expected to arrest the cell cycle at any given cell cycle phase. Adversely, conditional depletion of the Cdc14 is expected to stop the cell cycle at late mitosis, maintaining an active CDK.

**Figure 5:**
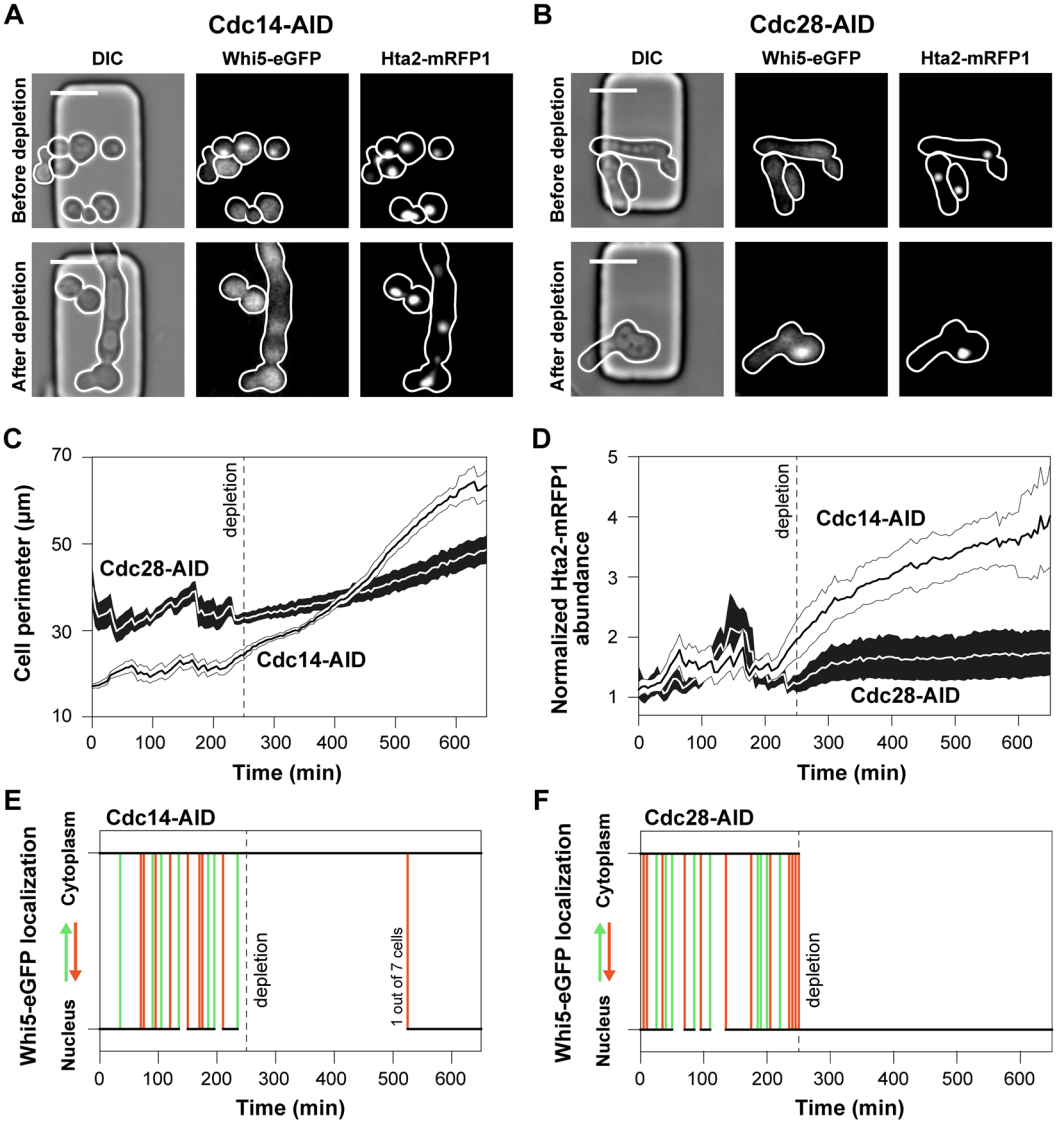
The auxin inducible protein degradation is efficiently used to deplete the Cdc14 and Cdc28 essential cell cycle regulators, and arrest the cell cycle at different phases. Microscopy images illustrate the phenotype of (**A**) Cdc14-AID and (**B**) Cdc28-AID cells prior and after protein depletion, including the cellular morphology, Whi5 localization and Hta2 abundance (scale 10 μm). In the RFP channel the number of genome copies is also visible. (**C**) The average perimeter of Cdc14-AID (white) and Cdc28-AID (black) cells (shaded area: SEM), manually measured in each single cell (7 cells for each strain), and each individual frame, using the white cellular borderline in the DIC channel (as in Fig. 5A,B), prior and after protein depletion (at 250 min). (**D**) The average intracellular histone abundance (shaded area: SEM) is plotted over time (before and after protein depletion) for the Cdc14-AID (white) and Cdc28-AID (black) strains (7 single cells each). The histone abundance of each single cell, was divided over the histone abundance just after the first cytokinesis in the G1 phase, which corresponds to one copy of the genome, to estimate the number of genomes per cell. The generated polyploidy after Cdc14-AID depletion is also visible in microscopy images and the number of Hta2-mRFP1 foci (Fig. 5A – Hta2-mRFP1). (**E**,**F**) The single cell Whi5 localization dynamics upon the depletion of (**E**) Cdc14-AID or (**F**) Cdc28-AID. When the trajectory becomes vertical there is Whi5 translocation from the nucleus to the cytoplasm (bottom to top – green lines) or from the cytoplasm to the nucleus (top to bottom – red lines). When the lines are horizontal (black lines), Whi5 remains in the cytoplasm (top) or the nucleus (bottom). Whi5 is a cell cycle inhibitor and reporter of CDK activity^48–50^. In the G1 phase it is localized in the nucleus. At START, Whi5 is phosphorylated by the active Cln3/CDK complex and is being sequestered into the cytoplasm signaling cell cycle initiation and the G1/S transition. Whi5 re-enters the nucleus at late mitosis. (**E**) After Cdc14-AID depletion (dashed vertical line) and halting of the late cell cycle, the anaphase promoting complex is not activated to degrade the remaining cyclins. Thus, the Cdc14-AID depleted single cells maintain a constitutive CDK activity (Whi5 in the cytoplasm). Spontaneous degradation of cyclins occasionally leads to cell cycle completion (1 out of 7 cells). (**F**) Adversely, depletion of Cdc28-AID depletion (dashed vertical line) immediately causes Whi5 sequestration into the nucleus, reporting absence of CDK activity.

Prior to the addition of auxin and protein depletion, the Cdc14-AID cells had normal morphology (Fig. 5A), in contrast to the Cdc28-AID cells, which exhibited constitutive filamentation (Fig. 5B). Because tagging of Cdc28 induced the filamentous phenotype in OsTIR1 expressing cells as well as wild type cells (Fig. S6), we conclude that the filamentation is not the result of basal Cdc28 depletion in the absence of auxin. Our results suggest that the degron tag disrupts the interaction of Cdc28 with its Cks1 regulatory domain, an interaction which reverses the Ras/PKA and Swe1 dependent filamentation^43^. Following the Cdc28-AID and Cdc14-AID cells after the depletion of their protein targets and cell cycle arrest, we found their size to increase (Fig. 5C). The Cdc14 depleted cells, increased in volume faster than the Cdc28 depleted cells, indicating early cell cycle activity and biomass synthesis even when the anaphase promoting complex and the late cell cycle are arrested. Consistently, through monitoring the abundance of histone Hta2, previously shown to correlate with DNA content^45, 46^, we found that Cdc14 cells also continued to replicate their DNA, with an approximate rate of one copy per 100 minutes (Fig. 5D). Adversely, DNA replication stalled in the absence of CDK activity (Cdc28 depletion).

Consistently with the CDK-centric model of cell cycle regulation^47^, our results confirm the sustained activity of the early cell cycle in cells lacking Cdc14: In the absence of APC activity, the late cyclins are not degraded and remain in the nucleus resulting in a sustained CDK activity, also reported by the cytosolic localization of Whi5^48–50^, an inhibitor of cell cycle transcription and phosphorylation target of Cdc28 (Fig. 5E). This sustained CDK-activity triggers biomass formation via the constitutive activation of the cell cycle transcription program, and DNA endo-replication cycles via the constitutive deactivation of the Sic1, an inhibitor of DNA replication^42, 50^. Adversely, in the absence of CDK activity (after Cdc28-AID depletion), reported by the immediate sequestration of Whi5-eGFP into the nucleus (Fig. 5F), cells cannot replicate their DNA.

The auxin-inducible protein depletion, exhibiting fast dynamics (approx. 25 min) well below the doubling time of yeast cells growing on glucose (approx. 100 min), was used successfully for to generate depletion phenotypes within a single generation or cell cycle phase. Thus, the AID system provides an alternative to the temperature-sensitive mutants, traditionally used to decipher the cell cycle machinery components^51^, but without their temperature-related effects on cellular physiology^5^.

## Discussion

Here, we comprehensively characterized the auxin-dependent degron system and its capabilities in single yeast cells. By providing optimal experimental parameters for the AID system and by revealing its advantages and potential off-target effects, our work adds value to a powerful tool for yeast researchers. The AID system allows for fast (25 min) protein depletion with minimal cell-to-cell variability. Exposing cells to 0.1 mM auxin for 20 min results in complete protein depletion. We found that the AID is reversible: after removal of the plant hormone, the recovery of the targeted protein starts immediately. However, to reach the equilibrium between protein synthesis and dilution, and to restore the original protein levels, several generations or growth cycles are necessary. The time required for complete recovery depends on the rate of protein expression, the protein stability, as well as its maturation time, and thus should be specifically determined for each protein target.

To avoid the photo-destruction of IAA during GFP excitation, leading to the production of toxic indole derivatives and growth defects, NAA should be used in microscopy experiments. As shown here, NAA at 0.1 mM has no effect on growth rate, while it induces protein depletion, similarly to IAA. Although the auxin inducible degron performs optimally when used to deplete essential proteins, or to generate binary phenotypes, it may also serve for the generation of quantitative growth phenotypes when the appropriate controls are used to discriminate between potential off-target effects and those specific to the depletion of the target protein.

Possibly the biggest advantage from combining microfluidics and single cell technologies with the auxin inducible degron is the capacity to assess intercellular variability. For instance, in cell cycle studies, the fact that cells are present at different cell cycle stages in a microfluidics device can be exploited for the generation of highly informative datasets: auxin reaches the cells at different cell cycle phases. As a result, the function of a targeted protein can be studied across the entire cell cycle in a single experiment. Similarly, the protein depletion phenotype may be assessed in young and aged cells, all growing in the same microfluidic device, under the exact same conditions, yielding information on age related biological processes. For instance, our finding that protein depletion is slower in newborn daughters as compared to their mother yeast cells (Fig. 1G and Movie S1), indicates that either protein synthesis rates are enhanced in daughter cells, or their ubiquitination and protein degradation machineries are not fully active as compared to mother cells.

Overall, the AID system is a simple and highly versatile system, providing excellent means to dynamically perturb biological systems. The characterization, insights and solutions provided in this work will allow yeast researchers to adapt this powerful tool for single cell perturbation experiments. We expect that - fueled by recent developments in microfluidics setups^26–31^ and in novel single cell reporters^52–54^, as well as the need to zoom into the single cell level – these types of experiments will gain importance.

## Methods

### Strains and strain construction

The prototrophic YSBN6 strain, derived from S288c, and its HIS^−^ variant (YSBN16) were used. For an overview on strains refer to Table S1.

In order to dynamically deplete Gcn5 we tagged the protein with a truncated version of the degron tag (AID^71–114^), previously tested and successfully applied in yeast^11^. Flanking sequences adjacent the stop codon of the Gcn5 coding sequence were amplified from the YSBN6 wild type genomic DNA (primer pairs GCN5-CDS and GCN5-DOWN – Table S2). The truncated degron tag (AID^71–114^) was amplified from the pNAT-AID*-9myc plasmid^11^ (Gcn5-IAA primer pair – Table S2). The NatMX expression cassette, offering resistance to clonNAT was amplified from the pAG36 plasmid^55^ (primer pair Gcn5-NatMX – Table S2). The fluorescent protein mCherry was amplified from the pBS35 plasmid^56^ (Gcn5-mCherry primer pair – Table S2). The ampicillin resistance cassette and the ColE1 origin of replication, used for selection and amplification into *Escherichia coli*, were amplified in one piece from the pC6B plasmid^46^ (Gcn5-Amp primer pair – Table S2). All six pieces were assembled into the pG4J plasmid using Gibson assembly. The pG4J plasmid, encoding for the Gcn5-AID tagging cassette, was verified using sequencing (primers Seq12, Seq24, Seq25, Seq26 – Table S2). The pG4J plasmid, was linearized (Gcn5-Lin primer pair – Table S2) and used to transform YSBN6.OsTIR1w/oGFP cells, under the selection of ClonNAT, for the construction of the YSBN6.OsTIR1w/oGFP.G4J strain (Table S1). We used lithium acetate high efficiency transformation protocol for all yeast transformations performed in this study. Correct integration of the Gcn5-AID cassette was confirmed using PCR (primer pairs Gcn5-Ver1 and Gcn5-Ver2 – Table S2).

To verify the Gcn5-AID conditional phenotype we constructed a Gcn5 deletion strain (YSBN6.Gcn5Δ – Table S1). The Gcn5 deletion cassette was amplified (primer pair GCN5-Del – Table S2) from the yeast deletion collection and the clone E21^57^ and was used to transform YSBN6 wild type cells, under the selection of G418. Correct integration was verified using PCR (GCN5-Del primers – Table S2).

The YSBN16.Whi5-eGFP.Hta2-mRFP1.OsTIR1w/oGFP.G23ARFPex strain (Table S1) was constructed in the following manner: The coding sequence of the OsTIR1 F-box protein was amplified from the pOsTIRw/oGFP plasmid^46^ (primers Seq 4 – Table S2), the Hta2-mRFP1 tagging cassette was amplified from the KOY.TM6*P hxk2-GFP hta2-mRFP1 strain^58^ (primers Hta2Lin – Table S2), and the Cdc14-AID tagging cassette was amplified from the pG23ARFPex plasmid^46^ (primers pG23ALinRFPex – Table S2). The three amplicons were used to transform YSBN16.Whi5-eGFP yeast cells^46^ (Table S1) under the selection of G418, phleomycin and ClonNAT respectively, in three separate genomic integration steps.

For the C-terminal tagging of Cdc28 with the degron sequence (AID^71–114^)^11^, the pG25 plasmid was constructed. The Cdc28 flanking sequences adjacent the stop codon were amplified (primer pairs Cdc28-CDS and Cdc28-DOWN – Table S2). The degron tag together with the NatMX antibiotic resistance cassette were amplified in one piece from the pG23ARFPex plasmid^46^, (IAA-NatMX primer pair – Table S2). The ampicillin resistance cassette and the ColE1 origin of replication, used for selection and amplification into *Escherichia coli*, were amplified in a single piece from the pC6B plasmid^46^ (primer pair Amp – Table S2). The four pieces were assembled into the pG25 plasmid, using Gibson assembly. Correct assembly was verified using sequencing (primers Seq103 and Seq104 – Table S2). The pG25 plasmid was linearized (Cdc28-Lin primer pair – Table S2) and was used to transform YSBN16.Whi5-eGFP.Hta2-mRFP1.OsTIRw/oGFP cells (Table S1), under the selection of ClonNAT for the construction of the YSBN16.Whi5-eGFP.Hta2-mRFP1.OsTIRw/oGFP.Cdc28-AID strain (Table S1). Correct integration was verified using PCR (Cdc28-Lin primers – Table S2).

### Cultivation

All experiments were performed on minimal medium^33^ with 10 gL^−1^ glucose. The pH of the minimal medium was adjusted with 10 mM K-Phthalate-KOH (pH 5) at a final pH of 5.1 or with 100 mM KH_2_PO_4_-KOH (pH 7) at a final pH of 6.8, as specified in the text. The medium adjusted to pH 6.8 was prepared before every experiment to avoid salt precipitation. Single yeast colonies growing on YPD 20 gL^−1^ glucose agar plates were used to inoculate 10 mL of minimal 10 gL^−1^ glucose medium in 100 mL shake flasks, and grown (at 30 °C, 300 rpm) overnight. The overnight culture was diluted in fresh medium to an OD of 0.1, and grown to an OD between 1 and 1.5 (exponential growth on 10 gL^−1^ glucose).

The exponentially growing cells were diluted to an OD of 0.05 and loaded into the microfluidic chip as described^26, 27^. Cells were fed with minimal 10 gL^−1^ glucose medium. The provided medium was pre-warmed to 30°C and saturated with atmospheric air by shaking at 300 rpm for at least two hours prior to use. Our microfluidic experimental set-ups are described in detail in Fig. S1A and B.

In the microfluidic device the Gcn5-AID, Cdc14-AID and Cdc28-AID strains (Table S1) were mixed with control cells expressing mGFP-AID (YSBN6.G2J – Table S1 ref. 46), to monitor the time when auxin reaches the cells, and to compare their depletion and recovery dynamics. For the flow-cytometry experiments with batch cultures, exponentially growing cells were diluted in fresh, pre-warmed and air saturated minimal medium containing 10 gL^−1^ glucose, at an initial OD of 0.1.

### Microscopy

Image acquisition (Nikon Ti-E inverted microscope with an Andor 897 Ultra EX2 EM-CCD camera; CoolLed pE2 excitation system; Nikon PFS dynamic focusing system) took place every 5 min, in the DIC and fluorescence channels using a 40x Nikon Super Fluor Apochromat objective. For GFP measurements (mGFP-AID and Whi5-eGFP), cells were excited at 470 nm with 15% light intensity using a 470/40 nm bandpass filter. The GFP emission was recorded using a 495 nm beamsplitter and a 525/50 nm emission filter. For the RFP measurements (Hta2-mRFP1 and Gcn5-mCherry-AID), cells were excited at 565 nm using a 560/40 nm bandpass filter. The mCherry emission was recorded using a 585 nm beamsplitter and a 630/75 nm emission filter. 20% LED power was used for the excitation of mRFP1 (fused with Hta2) and 50% for the excitation of mCherry (fused with Gcn5). No binning or EM gain was applied. 200 msec exposure time was applied for recording the mGFP and mRFP1 fluorescence, as well as during DIC imaging. 600 msec exposure time was applied for mCherry imaging. In the DIC channel, a halogen lamp was used as a light source, the light of which was filtered through an ultraviolet light filter (420 nm beamsplitter) to minimize cell damage during the long image acquisition. The NIS elements software (LIM) was used to control the microscope and for image acquisition.

### Image Analysis

For segmentation and tracking of single yeast cells, the BudJ plug-in^49^ for ImageJ^59^ was used. Specifically, cells were selected by clicking in the center, and they were automatically segmented and tracked based on the pixel intensity change at the perimeter of the cell in the DIC images. The average fluorescence intensity of the pixels contained within the specified cell boundaries was determined for each cell and fluorescence channel (GFP and/or RFP). The modal grey value corresponding to the background fluorescence of the whole field of view in each fluorescent channel was subtracted from the fluorescence of each monitored cell, at each time point.

For the quantification of the nuclear concentration of Gcn5-mCherry-AID and its dynamic depletion upon the addition of auxin, we used the BudJ plug-in^49^ for ImageJ^59^ and specifically the *Cluster Index* function. We set the cluster threshold at 1 standard deviation (brightest pixels by 1 standard deviation at the foci), the minimum fluorescence change at foci at 5%, the minimum foci size at 2 pixels, and the maximum foci size at 10 pixels. The average cytoplasmic RFP auto-fluorescence, measured in the control cells (mGFP-AID), which did not express RFP, was subtracted from the nuclear Gcn5-mCherry-AID fluorescence.

The conditionally or constitutively filamentous Cdc14-AID and Cdc28-AID cells, were manually segmented on the basis of the cellular borderline in the DIC channel, using ImageJ^59^. For the quantification of the intracellular Hta2-mRFP1 abundance, the whole cell median fluorescence was used as the cellular background fluorescence, and subtracted from the fluorescence images. The total fluorescence, corresponding to the DNA abundance, was quantified by multiplying the segmented whole cell area in μm^2^ (number of scaled pixels: 0.4 μm per pixel – 40x objective), by the mean fluorescence intensity. The nuclear or cytoplasmic localization of Whi5 was manually determined for each single cell.

### Flow cytometry

A BD Accuri flow-cytometer was used to record the dynamics of mGFP-AID depletion upon the addition of 0.5 mM of auxin. Exponentially growing cells on 10 gL^−1^ glucose, were diluted using the same medium at an OD of 0.2 and a final volume of 1.8 mL, and sampled continuously using 10 µl/min flow-rate, and 8 µm core size (diameter of sample flow in between the sheath laminar flow). The plant hormone was added to a concentration of 0.5 mM to the sample without stopping flow and gently mixed by pipetting.

The same instrument was used to measure the cell count of exponentially growing cells with mGFP-AID, Cdc14-AID and Cdc28-AID upon the addition of auxin. Appropriate dilutions were applied to maintain the cell count below 200000 cells/25 μl.

### Data availability

The data generated during and/or analyzed during the current study are available from the corresponding author on reasonable request.

## Electronic supplementary material

Supplementary information

Movie S1
