# Supplementary material for: Quantitative characterization of the auxin-inducible degron: a guide for dynamic protein depletion in single yeast cells
Source: Sci Rep. 2017 Jul 5;7:4704. doi: 10.1038/s41598-017-04791-6 (PMC5498663; doi:10.1038/s41598-017-04791-6)
Supplement: Supplementary file 1 — Supplementary information [file 41598_2017_4791_MOESM1_ESM.pdf]

Supplementary Information for

Quantitative characterization of the auxin-inducible degron: a guide for dynamic protein depletion in single yeast cells

Alexandros Papagiannakis, Janeska J de Jonge, Zheng Zhang, Matthias Heinemann

Molecular Systems Biology, Groningen Biomolecular Sciences and Biotechnology Institute, University of Groningen, Nijenborgh 4, 9747 AG Groningen, The Netherlands;

Supplementary Figures 2
Figure S1: Single cell experimental set-ups used in this study. 3
Figure S2: The dynamics of targeted protein depletion across different auxin concentrations and environmental pH values. 4
Figure S3: Auxin concentration-dependent effects on the growth rate of yeast populations. 6
Figure S4: The growth of yeast mothers and their newborn daughters are equally affected by auxin. 7
Figure S5: The dynamics of protein depletion with NAA and IAA are similar when the applied concentrations are equal to or higher than 0.05 mM. 8
Figure S6: Tagging of Cdc28 with the degron sequence ($IAA^{71\text{-}114}$) causes constitutive filamentation even in the absence of OsTIR1. 9
Supplementary Movie 10
Movie S1: The auxin induced protein depletion is slower for the yeast daughters, as compared to their mothers. 10
Supplementary Tables 11
Table S1: Yeast strains developed and/or used in this study. 11
Table S2: Primer sequences used for the development of all recombinant strains. 13
Supplementary References 17

Supplementary Figures

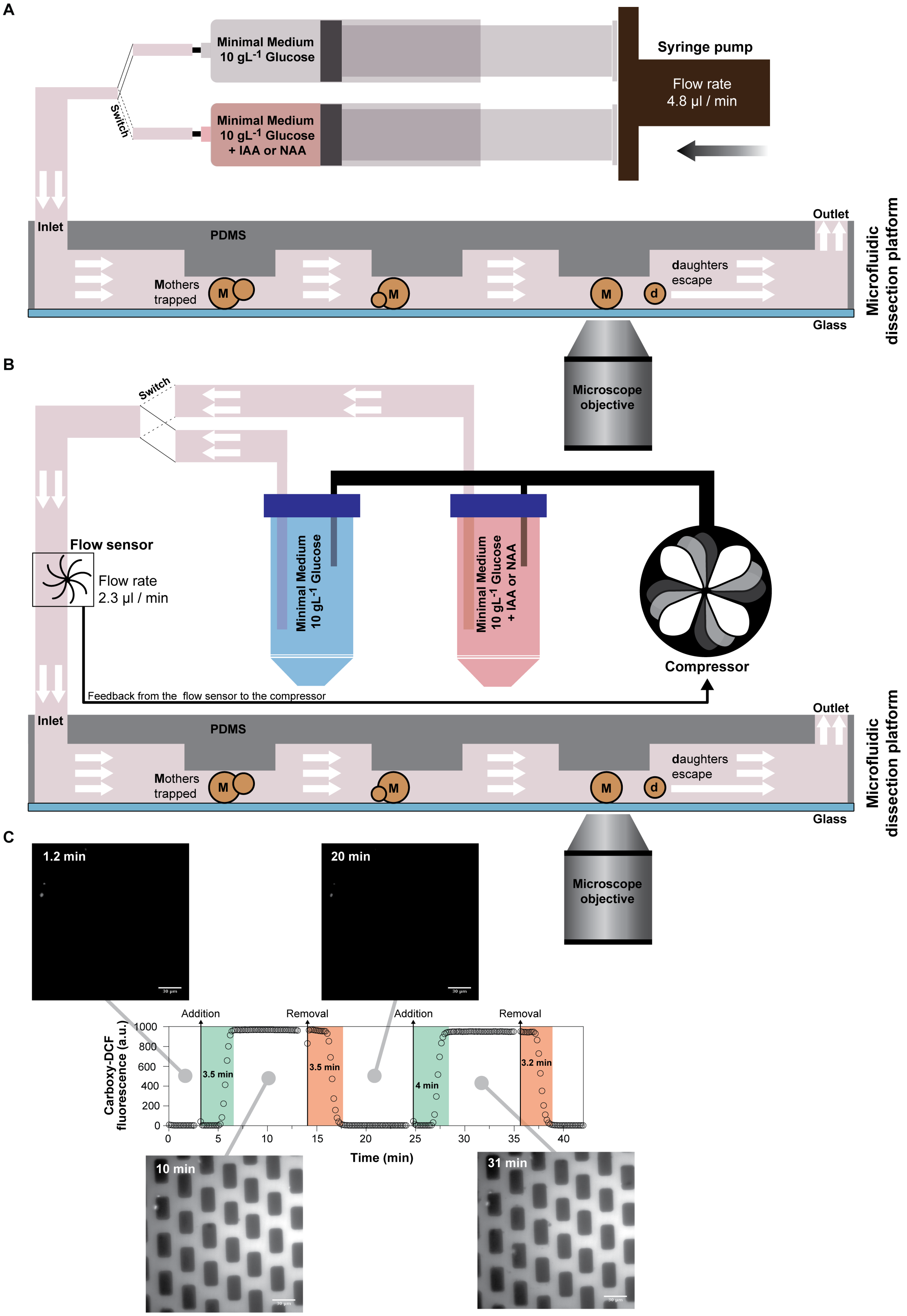

Figure S1: Single cell experimental set-ups used in this study.

Single yeast mothers are trapped in a PDMS (Polydimethylsiloxaan) based microfluidics dissection platform. The smaller daughter cells are washed away by constant medium flow, allowing for the microscopic observation of single yeast mothers over multiple generations. Information about the construction and use of the microfluidic device is available [1]. Medium is perfused through the microfluidics dissection platform **(A)** either using a programmable syringe pump (Harvard Apparatus – Standard Infuse/Withdraw 11 Elite) or **(B)** air-pressurized flow control (Everflow – OB1) together with flow sensors (Everflow – MFS2). **(C)** The dynamics of pressurized air-driven flow allowed for rapid medium switches (3 to 4 minutes) as shown in a control experiment, where we switched to minimal medium (10gL^{-1} glucose) containing 5-(and -6)-Carboxy-2,7-Dichlorofluorescein (Carboxy-DCF) and back to minimal medium (10gL^{-1} glucose) without the dye, in the microfluidic dissection platform. The fluorescence was monitored every 10 seconds in the GFP channel. The pressurized air-driven flow was exclusively used in the auxin-pulse experiments (Fig. 3).

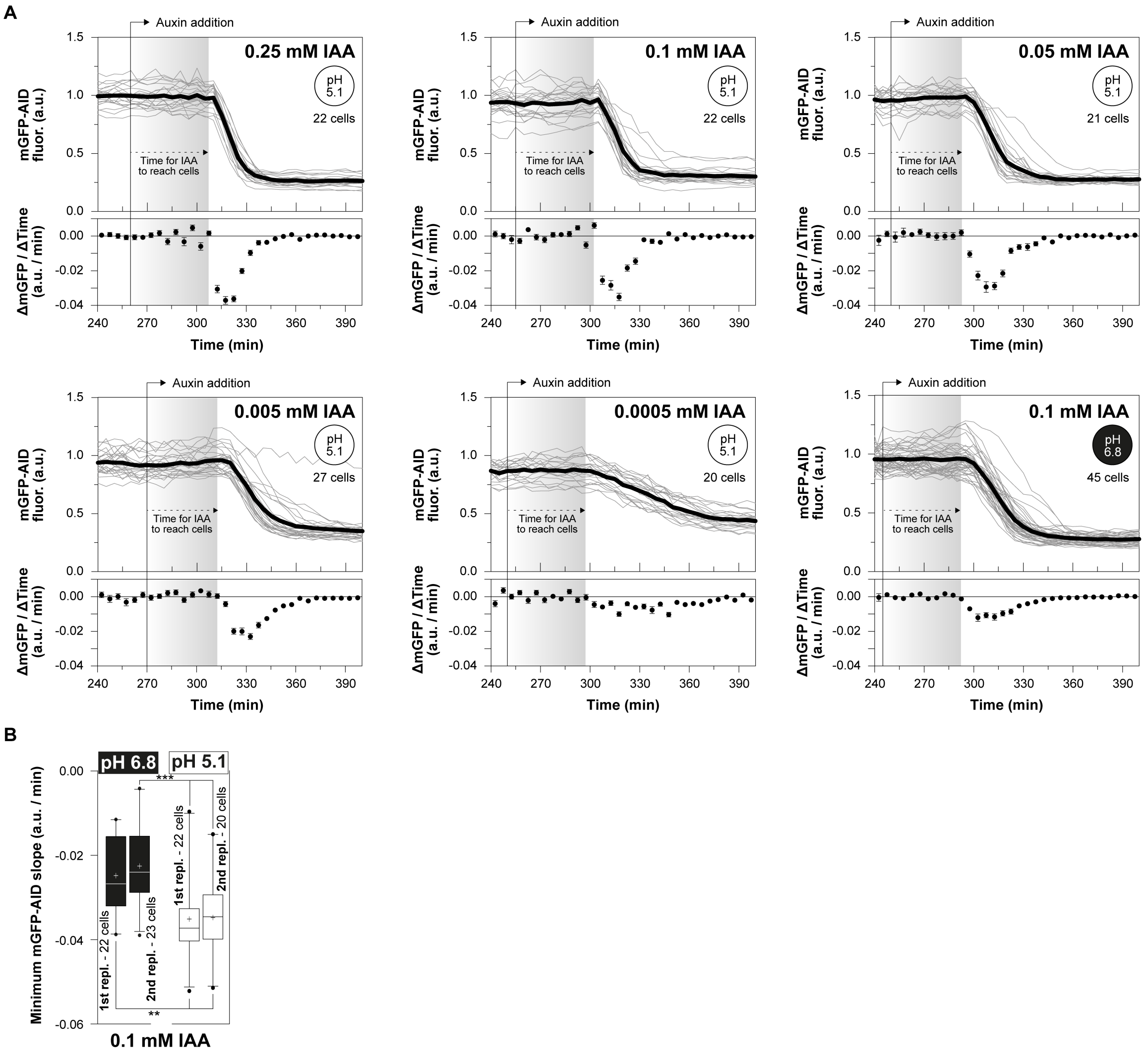

Figure S2: The dynamics of targeted protein depletion across different auxin concentrations and environmental pH values.

(A) Top figures: the average (black lines) and single cell (grey lines) mGFP-AID trajectories are presented for each auxin concentration and environmental pH value (5.1 or 6.8), as in Fig. 1B-C. Each single cell trajectory was normalized by dividing over the mean mGFP-AID signal from 0 to 240 minutes. Bottom figures: average rate (ΔmGFP/ΔTime) of mGFP-AID depletion (error bars: SEM). The minimal ΔmGFP/ΔTime values (bottom figures), or maximum rates of mGFP-AID depletion, across auxin concentrations and extracellular pH valiues are also presented in Fig. 1D. A comparison between the single cell mGFP-AID levels before and after auxin addition (top figures), was applied to estimated the completeness of mGFP-AID depletion in Fig. 1E. The deviation between the single cell mGFP-AID trajectories for each given auxin concentration (top figures) was used to estimate the uniformity of protein depletion in Fig. 3F. **(B)** The minimum

mGFP-AID slope, or the maximum rate of mGFP-AID depletion, was estimated (as in Fig. 1D), for the same auxin concentration (0.1 mM), at pH 6.8 (grey boxes) or pH 5.1 (white boxes) (error bars: 5–95 percentiles, cross: mean). Lower pH significantly increases the rate of mGFP-AID depletion (Kruskal-Wallis test, Dunns post-test for selected pairs of data, $p < 0.0001$ ***, $p < 0.001$ **).

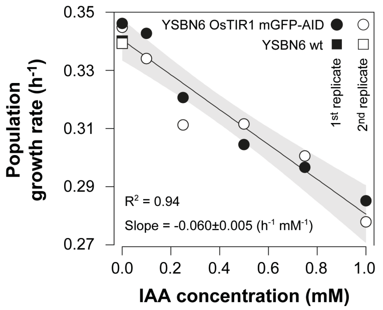

Figure S3: Auxin concentration-dependent effects on the growth rate of yeast populations. The growth rate was measured on the population level using flow cytometry (logarithmic increase of cell count over time), on different concentrations of the plant hormone (0 to 1 mM).

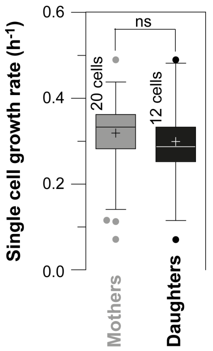

Figure S4: The growth of yeast mothers and their newborn daughters are equally affected by auxin.

The single cell growth rate distributions for yeast mothers (grey box) and newborn daughter (black box) after the addition of 0.1 mM auxin (Error bars: 5–95 percentiles, cross: mean) are not significantly different (Mann-Whitney test, $p > 0.05$ ns).

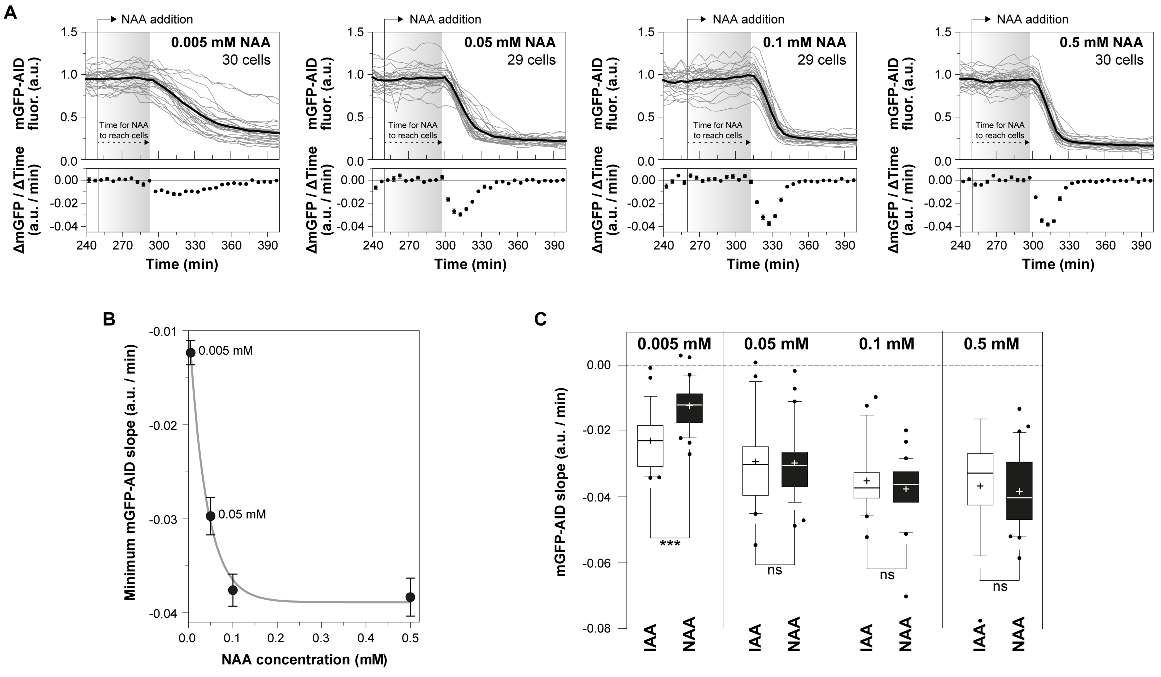

Figure S5: The dynamics of protein depletion with NAA and IAA are similar when the applied concentrations are equal to or higher than 0.05 mM.

(A) Top figures: the average (black lines) and single cell (grey lines) mGFP-AID trajectories are presented for each NAA concentration and environmental, as in Fig. 1B-C. Each single cell trajectory was normalized by dividing over the mean mGFP-AID signal from 0 to 240 minutes. Bottom figures: average rate (ΔmGFP/ΔTime) of mGFP-AID depletion (error bars: SEM). **(B)** The minimal ΔmGFP/ΔTime values, or maximum rates of mGFP-AID depletion, across NAA concentrations (error bars: SEM, same data as in Fig. S5A – bottom figures). A two-phase exponential decay function was fitted (y-intercept set to zero). **(C)** Comparison of the mGFP-AID depletion dynamics after the addition of IAA (white boxes) or NAA (black boxes) (Error bars: 10–90 percentiles, cross: mean). We tested different concentrations (0.005mM, 0.05 mM, 0.1 mM, 0.5 mM) of auxin (same data as in Fig. S2A and 1B) and its synthetic alternative NAA (same data as in Fig. S5A). We found identical depletion dynamics (Mann-Whitney test, $p > 0.05$ ns) for concentrations equal or higher to 0.05 mM. For lower concentrations (0.005 mM) we found the NAA to induce a slower protein depletion in comparison to IAA (Mann-Whitney test, $p < 0.0001$ ***). Our results indicate that the NAA has a lower binding affinity to the OsTIR1 protein than IAA. Given the unspecific growth defects of IAA when combined with blue light illumination and GFP excitation, and the similar protein depletion dynamics induced by (≥ 0.05 mM) IAA and NAA, we recommend the use of NAA in microscopy experiments.

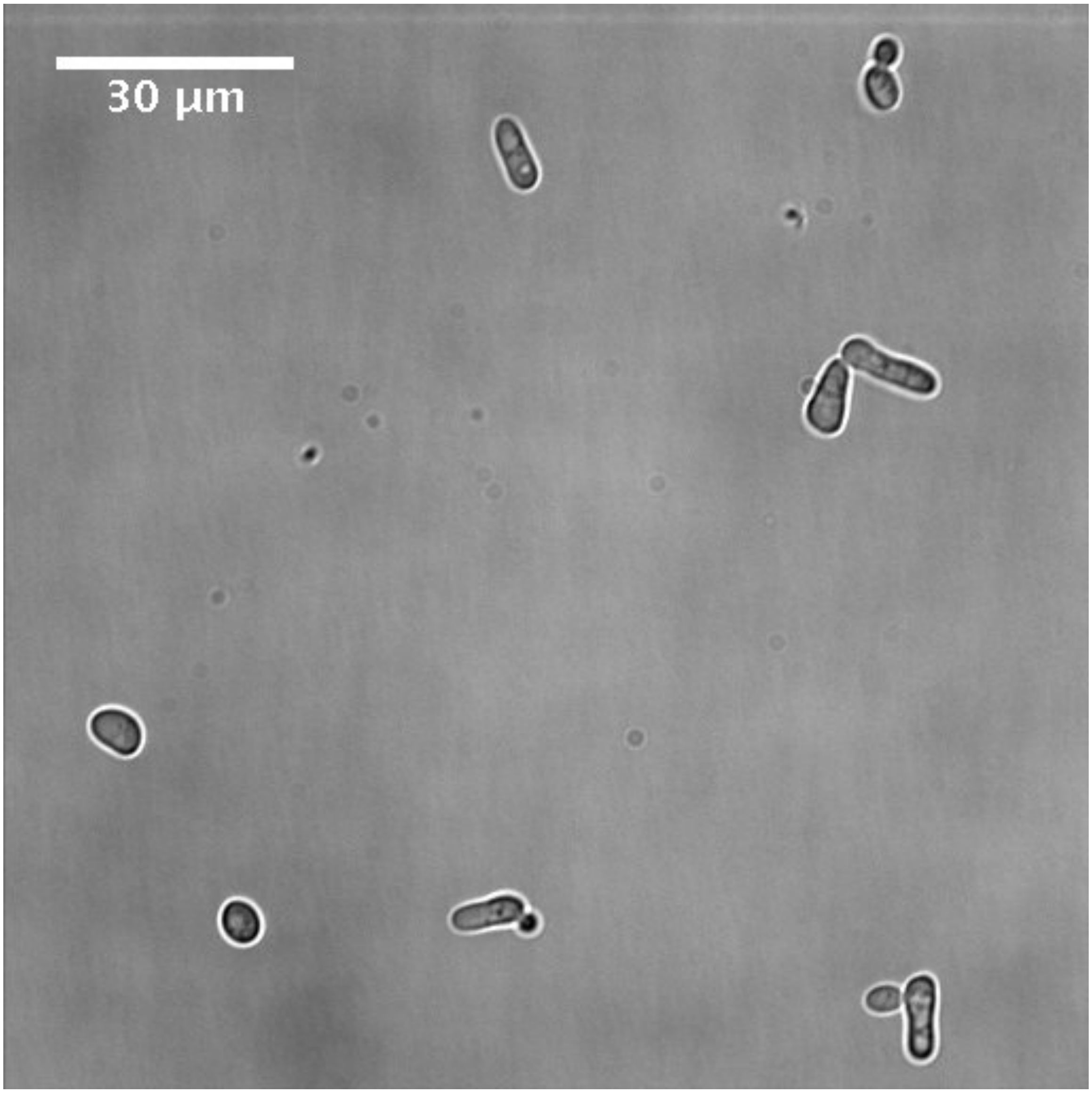

Figure S6: Tagging of Cdc28 with the degron sequence ($IAA^{71\text{-}114}$) causes constitutive filamentation even in the absence of OsTIR1.

Exponentially growing YSBN6 Cdc28-AID cells (YSBN6.G25ARFPex – Table S1) in minimal medium supplemented with $10gL^{-1}$ glucose, in the absence of auxin.

Supplementary Movie

Movie S1: The auxin induced protein depletion is slower for the yeast daughters, as compared to their mothers.

Yeast cells expressing mGFP-AID, and trapped in the microfluidic device are presented (scale 10 μm). The DIC and GFP channels have been merged. 2 daughter cells are born at 04:00 hh:mm and 04:40 hh:mm just prior the auxin inducible protein degradation (starting at 05:00 hh:mm), exhibit slower protein depletion dynamics. At 05:30 hh:mm there are no traces of the mGFP-AID protein in the mother cells. However, it takes 30 to 40 minites longer for the two newborn daughters to fully deplete their mGFP-AID fluorescence signal (at 06:00 and 06:10 hh:mm respectively).

Supplementary Tables

Table S1: Yeast strains developed and/or used in this study.

Strain	Genotype	Source
YSBN6	YSBN6 *wild type*	Steve Oliver lab, Cambridge
YSBN16	YSBN6 *his3Δ1*	[2]
YSBN16 Whi5-GFP	YSBN16<br>Whi5::eGFP-HIS3MX6	[3]
YSBN6.C6B	YSBN6<br>HO::TEF1p-TetR-iPFY1p-eGFP-KanMX4	[3]
YSBN6.G2J	YSBN6<br>ho::ADH1p-OsTIR1-TEF1p-mGFP-AID-KanMX4	[3]
YSBN6.OsTIR1w/oGFP	YSBN6<br>ho::ADH1p-OsTIR1-KanMX4	[3]
YSBN6.OsTIR1w/oGFP.G4J	YSBN16<br>ho::ADH1p-OsTIR1-KanMX4<br>Gcn5::mCherry-AID$^{71-114}$-NatMX	This study
YSBN6.Gcn5Δ	YSBN6<br>Gcn5::KanMX4	This study
KOY.TM6*P hxk2-GFP hta2-mRFP1	KOY.TM6<br>Hta2::mRFP1-Ble<br>Hxk2::eGFP-KanMX4	[4]

YSBN16.Whi5-eGFP.Hta2-mRFP1.OsTIR1w/oGFP.G23ARFPex	YSBN16<br>ho::ADH1p-OsTIR1-KanMX4<br>Whi5::eGFP-HIS3MX6<br>Hta2::mRFP1-Ble<br>Cdc14::mCherry-AID$^{71-114}$-NatMX	This study
YSBN16.Whi5-eGFP.Hta2-mRFP1.OsTIR1w/oGFP.G25ARFPex	YSBN16<br>ho::ADH1p-OsTIR1-KanMX4<br>Whi5::eGFP-HIS3MX6<br>Hta2::mRFP1-Ble<br>Cdc28::AID $^{71-114}$-NatMX (C-terminal tagging)	This study
YSBN6<br>G25ARFPex	YSBN6<br>Cdc28::AID $^{71-114}$-NatMX (C-terminal tagging)	This study

Table S2: Primer sequences used for the development of all recombinant strains.

The underlined sequences correspond to the overhangs designed for Gibson assembly. The term "gDNA" stands for purified genomic DNA.

Primer	Fwd/Rev	Sequence (5′ to 3′)	Templates
GCN5 CDS	Fwd	GCGGCTCGTATGTTGTGTGGCGAAATCGCATATTGTAAGG	YSBN6 wild type gDNA
	Rev	TCCTCGCCCTTGCTCACCATATCAATAAGGTGAGAATATTCAGG	
GCN5 DOWN	Fwd	CGGCGGGGACAAGGCAAGCTTGCGTAGAAGAAGCTTTTCC	YSBN6 wild type gDNA
	Rev	GGAACAAGAGTCCACTATTATTAACTTTGAAAGAAGCTGAGC	
Gcn5 mCherry	Fwd	AATATTCTCACCTTATTGATATGGTGAGCAAGGGCGA	pBS35
	Rev	CCGGCGCCTGCACCGTCGACCTTGTACAGCTCGTCCATGC	
Gcn5 IAA	Fwd	GCATGGACGAGCTGTACAAGGTCGACGGTGCAGG	#2189 plasmid [5]
	Rev	ACGCCGCCATCCAGTGTCGACGTACGCTGAGCTGGA	
Gcn5 NatMX	Fwd	AAGATCCAGCTCAGCGTACGTCGACACTGGATGGC	pAG36 plasmid [6]
	Rev	GGAAAAGCTTCTTCTACGCAAGCTTGCCTTGTCCC	

Gcn5 Amp	Fwd	TCAGCTTCTTTCAAAGTTAATAATAGTGGACTCTTGTTCC	pC6B plasmid [3]
	Rev	CCTTACAATATGCGATTTCGCCACACAACATACGAGC	
Gcn5 Lin	Fwd	CGAAATCGCATATTGTAAGG	pG4J (This study)
	Rev	TTAACTTTGAAAGAAGCTGAG	
Cdc28 CDS	Fwd	TTATGCTTCCGCGGCTCGTATGTTGTGTGG GGTATTGCATACTGCCACTC	YSBN6 wild type gDNA
	Rev	AGCACCAGCCCCGGCGCCTGCACCGTCGACTGATTCTTGGAAGTAGGGGT	
Cdc28 DOWN	Fwd	CCGGGTGACCCGGCGGGGACAAGGCAAGCTCAAATGCTACTGCACTGTC	
	Rev	GTTCCAGTTTGGAACAAGAGTCCACTATTACGGTGACAATGAAACTCTTC	
IAA-NatMX	Fwd	GTCGACGGTGCAGG	pG23A [3]
	Rev	AGCTTGCCTTGTCCC	
Amp	Fwd	CCACACAACATACGAGC	
	Rev	TAATAGTGGACTCTTGTTCC	
NatMX	Fwd	ACAGTGGAAAATAGCTCGACACTGGATGGC	pG23A [3]
	Rev	GGAGTAGAAACATTTTGAAGCTATGGTGTGAGCTTGCCTTGTCCC	

Cdc28 Lin	Fwd	GGTATTGCATACTGCCACTC	pG25 (This study)
	Rev	CGGTGACAATGAAACTCTTC	
Amp	Fwd	TAATAGTGGACTCTTGTTCC	pG2J plasmid (This study)
	Rev	CCACACAACATACGAGC	
Hta2Lin	Fwd	CAATTTCGACTGCATGATGC	KOY.TM6*P hxk2-GFP hta2-mRFP1 gDNA [4]
	Rev	GGAAGAACTACGCACTCTTTC	
GCN5 Del	Fwd	AATGAGATGTAGCAATGCAG	E21 clone – Yeast Deletion Project [7]
	Rev	TGCGGATGATGGTTATCAAC	
GCN5 Ver1	Fwd	GGACGCAGGTAAGATTCTAT	YSBN6.OsTIR1 w/oGFP.G4J gDNA
	Rev	CCTTGGAGCCGTACATGAA	
GCN5 Ver2	Fwd	GACAGTCACATCATGCC	
	Rev	CCTCTGGTGAAGAAAGACT	
Seq25	Fwd	GGATAACCGTATTACCGCCT	pG4J (This study)
Seq12	Rev	CCTGAAGTCTAGGTCCCTAT	
Seq 24	Rev	GGTCACAGCTTGTCTGTAA	
Seq 26	Rev	GGTGATACCTTCACGAAC	
Seq 103	Fwd	ACCAAGTCTAGATCCACG	pG25 (This study)
Seq 104	Rev	TACGACCTCGGTAAATACG	
Cdc14LinRF	Fwd	GTCGACGGTGCAGG	pG23ARFPex [3]

Pex	Rev	TTTCTTGATGGAGCCACTT	
Seq 4	Fwd	AATTATCCTGGGCACGAG	pOsTIR1w/oGFP [3]
	Rev	ACTGTAAGATTCCGCCAC	

Supplementary References

1. Huberts, D. H. E. W. *et al*. Construction and use of a microfluidic dissection platform for long-term imaging of cellular processes in budding yeast. *Nat. Protoc.* **8,** 1019–1027 (2013).

2. Kümmel, A. *et al*. Differential glucose repression in common yeast strains in response to HXK2 deletion. *FEMS Yeast Res.* **10,** 322–32 (2010).

3. Papagiannakis, A., Niebel, B., Wit, E. C. & Heinemann, M. Autonomous metabolic oscillations robustly gate the early and the late cell cycle. *Mol. Cell* **65,** 285–295 (2017).

4. Schmidt, A. M. Flux-Signaling and Flux-Dependent Regulation in Saccharomyces Cerevisae. *PhD thesis, ETH Zurich* 1–154 (2014).

5. Morawska, M. & Ulrich, H. D. An expanded tool kit for the auxin-inducible degron system in budding yeast. *Yeast* **30,** 341–51 (2013).

6. Goldstein, A. L. & McCusker, J. H. Three new dominant drug resistance cassettes for gene disruption in Saccharomyces cerevisiae. *Yeast* **15,** 1541–53 (1999).

7. Winzeler, E. A. *et al*. Functional Characterization of the *S. cerevisiae* Genome by Gene Deletion and Parallel Analysis. *Science (80-.).* **285,** 901–906 (1999).
